# Different Outcomes of Acanthamoeba Keratitis: A Case Series

**DOI:** 10.7759/cureus.48129

**Published:** 2023-11-01

**Authors:** Hui Lin, Nurliza Khaliddin

**Affiliations:** 1 Department of Ophthalmology, University of Malaya, Kuala Lumpur, MYS

**Keywords:** treatment, contact lens, outcome, keratitis, acanthamoeba

## Abstract

*Acanthamoeba* keratitis is a potentially sight-threatening infection of the cornea that can lead to blindness. Over the past few decades, there has been a rise in the reported cases worldwide, which is in line with the increase of contact lens wearers. Here, we present three cases of *Acanthamoeba* keratitis with different outcomes.

## Introduction

*Acanthamoeba* keratitis was first reported in the 1970s. In recent years, the number of reported cases has been increasing, year after year at an alarming rate. Although the incidence of *Acanthamoeba* keratitis is way less than bacterial and viral keratitis, it is still one of the most dreadful and sight-threatening corneal infections that can lead to blindness. One of the most common risk factors for the development of *Acanthamoeba* keratitis is contact lenses. Contact lenses are used not only for cosmetic purposes but in orthokeratology as well [1]. The influx of contact lens wearers, easy access to purchasing cheap contact lenses, and poor contact lens hygiene, all lead to the emergence of this infection worldwide. On the other hand, non-contact lens wearers can develop *Acanthamoeba* keratitis via trauma to the cornea as reported by Sharma et al. [2]. In this case series, we present three cases of *Acanthamoeba* keratitis with different outcomes.

## Case presentation

Case 1

A 41-year-old Malay lady was referred to our clinic for red, painless, and progressive blurring of vision in the right eye. She is a regular contact lens wearer with a history of poor hygiene in handling contact lenses. Initially, she was treated for herpetic keratitis for one month, but it was not responding to the treatment, which caused her vision to deteriorate further.

**Figure 1:**
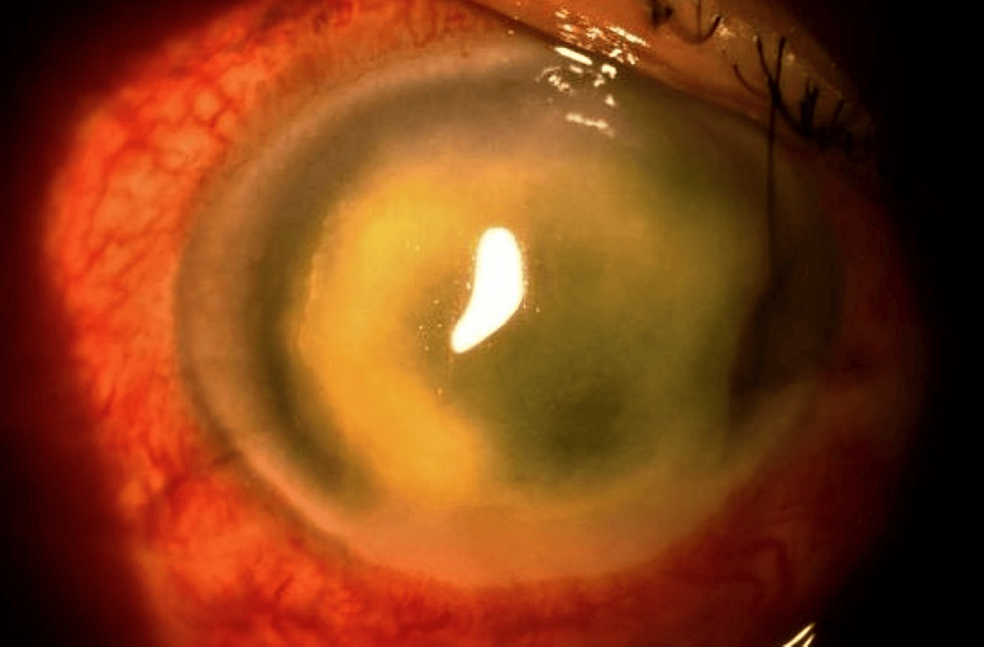
Case 1: Acanthamoeba keratitis

The patient’s presenting visual acuity was counting finger (CF) and 6/9 in the right and left eye, respectively. An ophthalmic examination of the right eye revealed injected conjunctiva and large central corneal ring infiltrates with perineuritis at the 6 o’clock position (Figure 1). B scan showed a clear vitreous, no loculation, and the retina was flat. Intraocular pressure was normal in both eyes. The left eye findings were unremarkable.

**Figure 2:**
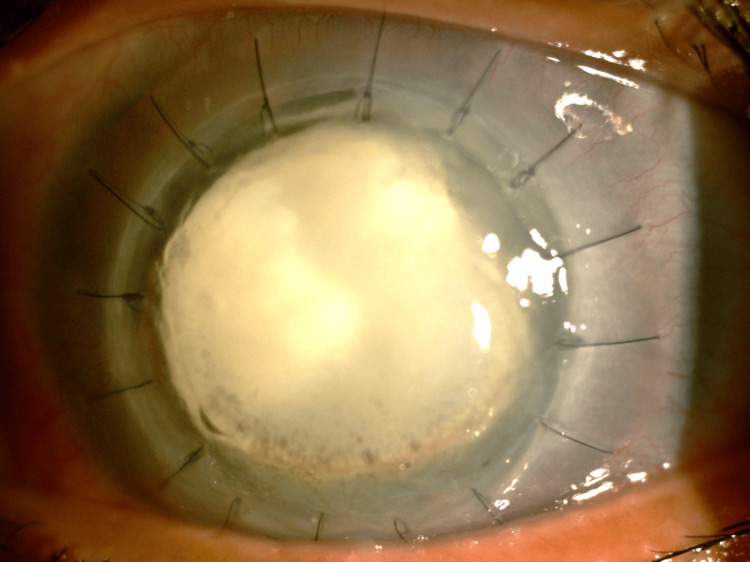
Penetrating keratoplasty

She was immediately started on topical chlorhexidine 1 hourly, topical Brolene 1 hourly, and topical levofloxacin 4 hourly on the right eye. With all the intensive treatment on board for about a month, the infection was not responding to the medical treatment and her eye condition deteriorated. Further examination showed a central corneal stromal abscess, a 4 mm level of hypopyon mixed with hyphema, and high intraocular pressure. Thus, therapeutic penetrating keratoplasty was performed and histopathological examination of the recipient cornea showed the presence of *Acanthamoeba* cysts in the stroma (Figure 2). Eight weeks after penetrating keratoplasty, she developed a recurrence of the infection. The grafted cornea was melting with corneal perforation. Hence, the right eye was eviscerated.

Case 2

A 30-year-old Malay lady was referred to our hospital for right eye pain, blurring of vision, and photophobia. She was initially treated for a right eye dendritic ulcer for two months, but her eye condition kept worsening for the past two weeks. She has been a regular contact lens wearer for the past six years and has good hygiene in handling her contact lenses.

**Figure 3:**
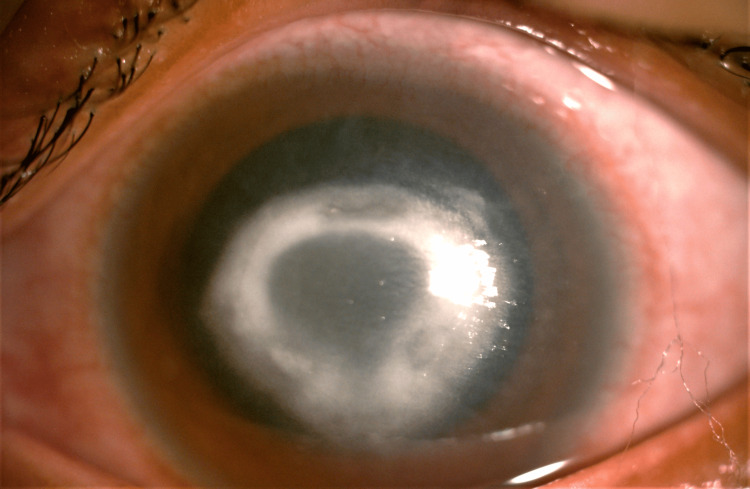
Case 2: Acanthamoeba keratitis

Vision of the right and left eye on presentation was 2/60 and 6/36 ph 6/24, respectively. The right eye showed conjunctival injection, edema in the cornea, and the presence of a central C-shaped ring infiltrate with an overlying epithelial defect, central endothelial plaque, perineuritis at 1 o’clock, and hypopyon level at the anterior chamber (Figure 3). B scan of the right eye showed no loculations and the retina was flat. The left eye findings were unremarkable. The intraocular pressures of both eyes were normal. Corneal scraping culture and sensitivity showed no growth.

She was treated for *Acanthamoeba* keratitis. Thus, topical Brolene, chlorhexidine, and antifungal were added to the topical antibiotics that were started earlier. After being on this treatment for about a month, her right eye showed signs of improvement. Further follow-ups revealed secondary high intraocular pressure on the right eye but was well controlled with two types of anti-glaucoma. Five months after treatment, scarring of the cornea with a best-corrected visual acuity of 6/36 was observed.

Case 3

A 32-year-old Malay lady was referred to our hospital for both eye redness and blurring of vision. She has been a contact lens wearer for the past 10 years, and her risk factors were prolonged wearing of contact lenses for more than 12 hours per day and having occasionally slept with her contact lenses on. Initially, she was treated for viral keratitis, and after three weeks, no improvement was seen.

**Figure 4:**
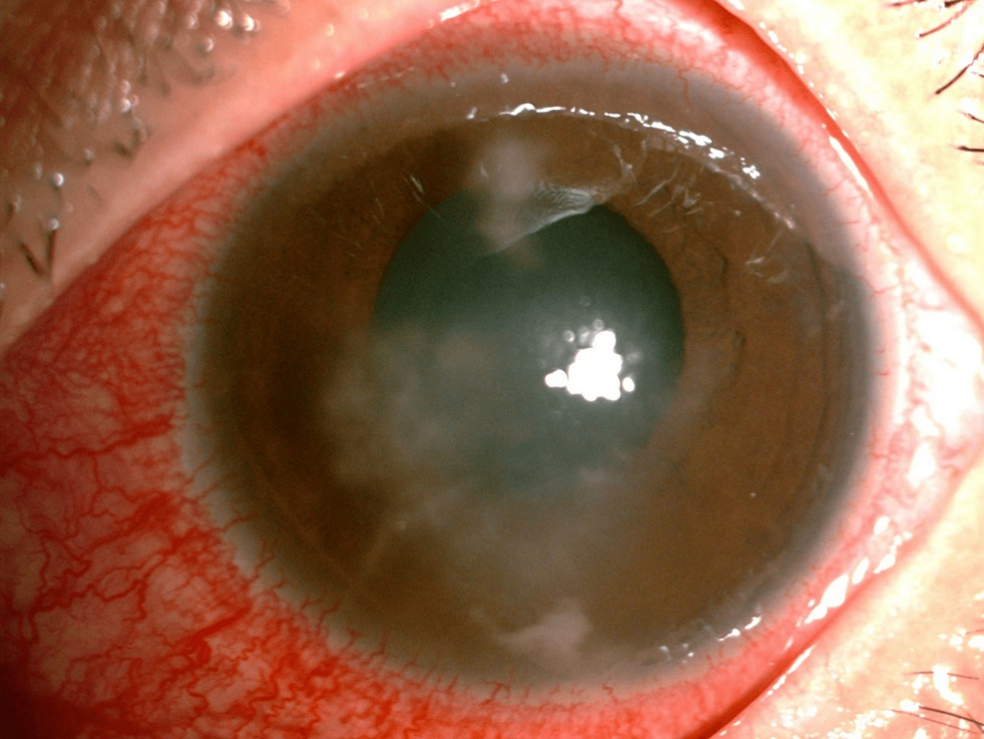
Right eye healing Acanthamoeba keratitis

**Figure 5:**
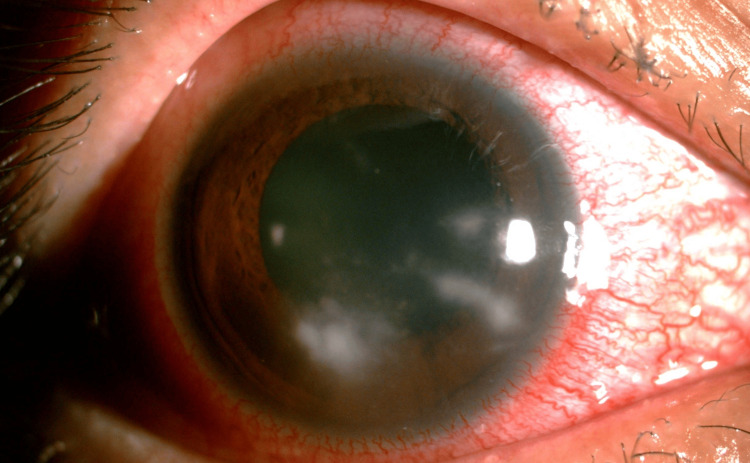
Left eye healing Acanthamoeba keratitis

Presenting vision was 6/12 for the right eye and 6/24 for the left eye. The right eye showed generalized conjunctival injection, perineural infiltrates at 3 and 4 o’clock positions, the presence of paracentral stromal infiltrates, and anterior chamber cells of 2+ (Figure 4). The left eye findings revealed generalized conjunctival injection, perineural infiltrate at the 7 o’clock position, the presence of discrete stromal infiltrates, and anterior chamber cells 2+ with fibrin (Figure 5). Intraocular pressure and fundus assessment were normal in both eyes. Corneal scraping for both eyes showed no growth.

She was treated for *Acanthamoeba* keratitis in both eyes. Topical Brolene, chlorhexidine, and topical antibiotic were started. Within two months, both eyes showed improvement with minimal scarring. Best-corrected visual acuity was 6/9 in both eyes.

## Discussion

*Acanthamoeba* keratitis is a potentially blinding infection of the cornea, caused by *Acanthamoeba* species. It was first described in 1973 [3-6]. The incidence in the United States is one to two cases per million contact lens wearers, and in the United Kingdom, it is reported to be 17-21 cases per million contact lens wearers. In Malaysia, the first *Acanthamoeba* keratitis case was reported in 1995 and a survey done from June 2002 to December 2002 by Kamel et al. showed that 9.1% (four of 44 cases) had culture positive for Acanthamoeba [7].

The most common risk factor for the development of *Acanthamoeba* keratitis is contact lenses. It is reported that in about 80-86% of the cases, *Acanthamoeba* keratitis is related to contact lenses [8-10]. Additional risk factors were poor compliance with contact lens hygiene, swimming while wearing contact lenses, cleaning the lens case with tap water, and exposure to contaminated water. On the other hand, in India, it is reported that the majority of *Acanthamoeba* keratitis is trauma-related. All three case series presented here had a similar risk factor, which is contact lens. In case 1, there is an additional risk factor, which is poor contact lens hygiene, whereas, case 3 had extended wear of contact lens.

It is difficult and challenging for an ophthalmologist to diagnose *Acanthamoeba* keratitis. Most of the time it is often mistaken for viral keratitis, thus leading to a delay in treatment. Early diagnosis and prompt medical treatment are crucial in securing a good visual prognosis. To diagnose *Acanthamoeba* keratitis, one should have a high clinical suspicion based on the history, clinical presentation, and clinical signs. All three cases were initially treated for viral keratitis, and they did not respond to the treatment that was given. Medical treatment for Acanthamoeba keratitis was delayed for case 1, case 2, and case 3 by one month, two weeks, and three weeks, respectively.

To date, there is no single drug that can treat *Acanthamoeba* keratitis. The recommended combined topical treatment is polyhexamethylene biguanide (PHMB) (0.02%) or chlorhexidine (0.02%) with or without propamidine 0.1% (Brolene) to be given every hour around the clock for the first few days. Treatment is then tapered based on clinical response, usually for 2-6 months. Furthermore, surgical treatment (corneal transplant) is another option to treat *Acanthamoeba* keratitis. It is not recommended to perform a corneal transplant in an acute condition as it is reported to have poorer results [11]. The success rate for corneal transplant is higher once the eye is quiet, and there is a minimum of three months discontinuation of medical treatment. However, if the infection worsens and topical or oral medications fail, there is a role in performing therapeutic keratoplasty. But there is a higher risk of graft failure and recurrence of the disease if surgery is performed in an inflamed eye.

All three cases had been started with chlorhexidine and Brolene. The patient in case 1 showed no response toward medical treatment and thus surgical treatment was opted for. Eight weeks after the corneal transplant, this patient developed a recurrence of the disease and evisceration was performed. In case 2 and case 3, both patients responded well to medical treatment. *Acanthamoeba* keratitis in case 2 healed with a significant corneal scar with a best-corrected visual acuity of 6/36. On the other hand, the patient in case 3 healed with minimal corneal scar with a best-corrected visual acuity of 6/9.

## Conclusions

*Acanthamoeba* keratitis remains a challenging disease to treat. With early detection and prompt treatment, a good visual outcome can be achieved. Besides, as the number of reported cases is on the rise due to increasing contact lens use for vision correction and cosmetic purposes, awareness should be created among the public on proper technique in handling contact lenses.
